# Crafting the Future of Community-Based Medical Rehabilitation: Exploring Optimal Models for Non-Inpatient Rehabilitation Services through a Narrative Review

**DOI:** 10.3390/ijerph21101332

**Published:** 2024-10-08

**Authors:** Iuly Treger, Amit Kosto, Dor Vadas, Alan Friedman, Lena Lutsky, Leonid Kalichman

**Affiliations:** 1 Department of Physical Medicine and Rehabilitation, Soroka University Medical Center, Beer Sheva 84101, Israel; 2 Faculty of Health Sciences, Ben-Gurion University of the Negev, Beer Sheva 84105, Israel; 3 Nuffield Department of Primary Care Health Sciences, University of Oxford, Radcliffe Observatory Quarter, Oxford OX2 6GG, UK; 4 South Department Administration, Clalit Health Service, Beer Sheva 84101, Israel; lenalu@clalit.org.il; 5 Department of Physical Therapy, Recanati School for Community Health Professions, Faculty of Health Sciences, Ben-Gurion University of the Negev, Beer Sheva 84105, Israel

**Keywords:** community-based medical rehabilitation, outpatient rehabilitation, multidisciplinary rehabilitation, physical medicine and rehabilitation physician

## Abstract

Community-based medical rehabilitation encompasses diverse programs that cater to patients outside of inpatient settings, such as home rehabilitation, day rehabilitation centers, and ambulatory clinics. While inpatient rehabilitation principles are widely standardized, outpatient programs display significant variability influenced by healthcare models, local environments, economic constraints, and available resources. This narrative review aims to explore and synthesize the various models of non-inpatient rehabilitation services, evaluating their effectiveness, cost-efficiency, and patient satisfaction. The review also seeks to identify optimal practices and strategies to enhance community-based rehabilitation, alleviate the burden on inpatient facilities, and improve patient outcomes through multidisciplinary and patient-centered approaches. Additionally, the study examines the critical role of a professional program coordinator and the importance of effective clinical communication in outpatient rehabilitation. A comprehensive search of peer-reviewed literature was conducted across multiple databases, focusing on studies that examined community-based rehabilitation models. The findings suggest that community-based rehabilitation programs are generally more cost-effective than inpatient programs, with their success being heavily dependent on the intensity and timing of interventions. Multidisciplinary approaches and high-intensity rehabilitation have shown promise in improving patient quality of life, though their effectiveness varies by condition. Despite limited research, the involvement of a Physical and Rehabilitation Medicine (PRM) physician as a program coordinator appears vital for ensuring continuity of care. Moreover, effective clinical communication is essential, impacting all aspects of patient care and interprofessional collaboration, with continuous adaptation required to meet the evolving needs of diverse patient populations.

## 1. Introduction

Medical rehabilitation is rapidly advancing in Israel and worldwide due to factors such as the aging population, improved outcomes of medical care, and heightened awareness of the significance of rehabilitation medicine [1]. The development of comprehensive outpatient rehabilitation treatment is also progressing swiftly, serving as both a model for replacing hospitalization and as an optimal professional intervention following early discharge from an inpatient program.

Outpatient medical care, often referred to as ambulatory care, entails services provided to patients without admission to an inpatient hospital or for stays shorter than 24 h. In medical rehabilitation, various terms like outpatient rehabilitation, ambulatory rehabilitation, and rehabilitation in primary care have been used to describe non-inpatient services [2]. This encompasses a broad spectrum of existing rehabilitation programs, ranging from multidisciplinary individual rehabilitation projects simulating inpatient-like services to customized programs delivered by a single discipline [2].

Rehabilitation medicine has undergone rapid development in recent decades and is acknowledged by the World Health Organization (WHO) as one of the leading medical strategies of the 21st century [3]. Cieza et al. [4] reported a 63% increase in global demand for rehabilitation between 1990 and 2019, attributed to population aging, advancements in acute medical treatment, and a growing understanding of the importance of rehabilitation [5]. Outpatient rehabilitation is poised to meet this escalating demand by providing professional care without requiring additional inpatient beds. Even for severely injured patients requiring subacute inpatient rehabilitation, community-based medical rehabilitation programs enable early discharge to the subsequent stage of the Individual Rehabilitation Management Plan (IRMP) [6].

Brochures from different rehabilitation services often outline prerequisites for inclusion in outpatient rehabilitation programs, considering factors such as patients’ functional levels [7], severity of impairments, complexity of medical conditions [8], and environmental support. In 2010, the WHO issued guidelines on Community-based Rehabilitation (CBR) principles and services. Historically, CBR has served as a conduit for providing rehabilitation-focused services to individuals in economically disadvantaged nations, leveraging Indigenous community assets. While the concept of CBR has evolved into a broader developmental strategy, active involvement in delivering rehabilitation services at the grassroots level remains essential for CBR initiatives. Relying on specialized rehabilitation centers may not be necessary or feasible for a significant portion of the population, especially those in rural areas, where many interventions can be effectively initiated within the community [9].

The WHO’s compendium, titled “Guide on Community Training for Individuals with Disabilities”, elucidates rehabilitation activities that are amenable to execution within the community using locally available resources. According to WHO guidance, CBR programs can offer crucial support through home-based visits, fostering an environment conducive to the sustained continuation of rehabilitation pursuits aligning with the evolving needs of individuals with disabilities [9].

CBR programs can be understood through three primary models: medical, social, and community-based. The medical model focuses on disability and impairment as medical issues, emphasizing diagnosis, treatment, and management by healthcare professionals in clinical settings. In contrast, the social model views disability as a societal issue, advocating for the removal of societal barriers and promoting social inclusion through community involvement and empowerment. The community-based model integrates medical and social aspects, providing holistic care utilizing local resources and community support systems. This model involves a multidisciplinary team, including healthcare professionals and community members, and emphasizes sustainable, long-term support within the community. By combining these approaches, CBR programs aim to improve the quality of life for individuals with disabilities and promote their inclusion in society [10,11].

In locations with established community-embedded rehabilitation services, fostering and maintaining strong connections with referral centers providing specialized rehabilitation provisions is crucial. Recognizing that the needs of individuals with disabilities evolve over time, there may be a prolonged need for ongoing, periodic support. Achieving effective rehabilitation outcomes depends on the creation of robust partnerships among individuals dealing with disabilities, rehabilitation professionals, and community workers involved in CBR efforts [9]. The European Union of Rehabilitation Physicians (UEMS PRM—The European Union of Medical Specialists—Physical and Rehabilitation Medicine (PRM) Section) defined a systematic process involving representatives from most member countries, including Israel, through the Delphi process. This process established a list of the types of medical rehabilitation systems that were implemented within the community rehabilitation framework. Various systems include rehabilitation day hospitalization, home-based rehabilitation, treatment within rehabilitation clinics, and other modalities [12]. Day hospitals and rehabilitation clinics may be situated within rehabilitation departments, independent community systems, or specialized centers.

Despite the growing recognition of the importance of rehabilitation services, there remains a lack of comprehensive understanding of effective non-inpatient rehabilitation services and their impact on patient outcomes. Addressing these gaps is crucial for developing evidence-based guidelines and improving rehabilitation services for diverse populations.

The aim of this narrative review is to comprehensively explore the various models of non-inpatient rehabilitation services, including home-based rehabilitation, day rehabilitation centers, and ambulatory clinics. We seek to evaluate these models in terms of their effectiveness, cost-efficiency, and patient satisfaction. Furthermore, this study aims to identify and highlight optimal practices and strategies to enhance CBR, reduce the demand on inpatient facilities, and ultimately improve patient outcomes through multidisciplinary and patient-centered approaches. Additionally, we aim to examine the pivotal role of a professional program coordinator in ensuring the success of outpatient rehabilitation programs and to assess the impact of effective clinical communication on both patient care and interprofessional collaboration.

## 2. Methods

### 2.1. Literature Search Strategy

The literature search for this narrative review was conducted to explore the landscape of outpatient rehabilitation programs. The approach focused on synthesizing existing literature rather than exhaustive systematic searches. A comprehensive search was conducted across multiple databases, including PubMed/Medline, Google Scholar, CINAHL, and Web of Science. The search strategy employed a basic set of keywords, including “Outpatient”, “Community”, “Homebased”, “Rehabilitation”, and “Comparison”. Additional keywords were tailored to each thematic subtitle to ensure a comprehensive scope.

### 2.2. Data Collection Process

The data collection period spanned from January 2023 to March 2024. Authors D.V. and A.K. retrieved the data, ensuring that the most recent and relevant studies were included in the review. The initial search was conducted in January 2023, and subsequent searches were performed periodically until March 2024 to capture any newly published articles.

### 2.3. Resolution of Discrepancies

In cases of disagreement, a third researcher, L.K., was consulted to act as a referee. This referee reviewed the differing opinions and facilitated a discussion to reach a consensus. If no consensus was achieved, the article was excluded based on predefined exclusion criteria.

### 2.4. Study Selection Criteria

The authors predetermined the review’s subtitles, and the search strategy was based on the hierarchy of evidence in evidence-based medicine [13]. This approach aimed to aggregate literature and prioritize evidence of the highest level that could offer insights into the review’s research question. Studies were selected based on their relevance to the narrative synthesis of outpatient rehabilitation programs.

Inclusion criteria:

- Studies that contributed to understanding clinical and economic comparisons.
- Studies focusing on patient impairments, disciplinary involvement, and program oversight by rehabilitation specialists.
- Qualitative and quantitative studies providing insights into outpatient rehabilitation programs.

Exclusion criteria:

- Studies not available in English.
- Studies published before 2000.
- Non-peer-reviewed articles, such as case reports and opinion pieces.
- Studies focusing on inpatient rehabilitation or not directly related to community-based or outpatient rehabilitation.
- Studies lacking sufficient data.
- Studies without clear or measurable outcomes.

### 2.5. Data Extraction and Analysis

Data extraction focused on identifying key themes and findings across selected studies. The thematic analysis outlined by Braun and Clarke [14] was employed to synthesize qualitative and quantitative evidence and present a cohesive narrative. This method involved the following steps: (1) reading and re-reading the data to become deeply familiar with the content; (2) systematically noting features of the data relevant to the research question; (3) collating codes into potential themes, gathering all data relevant to each potential theme; (4) checking if the themes work in relation to the coded extracts and the entire data set; (5) ongoing analysis to refine the specifics of each theme and the overall story the analysis tells; (6) weaving together the analytic narrative and data extracts to tell the story of the data.

By adhering to these inclusion criteria and conducting a comprehensive literature search, the review aimed to gather the most relevant and robust evidence available on the topic. The narrative approach allowed for the integration of diverse evidence and perspectives on outpatient rehabilitation, providing a holistic view of current practices and outcomes.

## 3. Results

### 3.1. Articles Selection

The initial search yielded a total of 812 articles. After screening titles and abstracts for relevance, 122 articles were selected for full-text review. Following the application of inclusion and exclusion criteria, 45 articles were included in the final synthesis. This process ensured that only the most pertinent and high-quality studies were included in the narrative review.

A total of 48 papers were selected, some of which were used twice in accordance with the relevant chapter (Table 1).

### 3.2. Outpatient Rehabilitation Cost-Effectiveness and Patient Satisfaction

Studies consistently support the cost-effectiveness superiority of various outpatient rehabilitation programs over inpatient programs. A cost-effective cohort study from 2022 revealed that home-based rehabilitation programs outperformed center-based inpatient programs in 29 out of 32 European countries. This study contributed to Quality Adjusted Life Years while saving 237 million Euros in European health and social services [15]. Similar findings were demonstrated with stroke survivors in Canada [16]. A systematic review based on electronic databases comparing adult rehabilitation programs in several states [17] also determined home rehabilitation to be more cost-effective than inpatient rehabilitation programs.

Moreover, patient satisfaction with outpatient services replacing inpatient hospitalization is generally high. A survey-based study reflected that over 70% of patients reported satisfaction with such services [18]. Results of a randomized control trial (RCT) supported the continued use of home-based rehabilitation [19].

Patient satisfaction with these services was also evident in interviews with early-discharged patients [20]. Participants expressed gratitude for the opportunity to receive multidisciplinary services at their homes, stating, *“…I think it’s so much better being at home, when your friends can come and visit you, so you’re not bored all day, and you haven’t got that horrible food… you’re just in your own environment, your own home”.*

### 3.3. Reducing the Load from the Inpatient Wards

CBR can also serve as a viable solution for the shortage of hospital beds. In a recent review by Friedman et al., titled “The Last Bed, Whom to Admit” [1], they reported on a small inpatient rehabilitation unit at a large university medical center representing a vast socioeconomic range. The paper discussed ethical issues and decision-making processes regarding the lack of hospital beds in the rehabilitation ward for inpatient rehabilitation. CBR, particularly home rehabilitation programs, can help alleviate the burden on busy facilities. The prolonged wait for a rehabilitation bed not only causes suffering for patients and their families but also adversely affects rehabilitation outcomes.

In a narrative review from 2017 [21] about early rehabilitation for stroke patients, it was suggested that although the precise commencement timing for post-stroke rehabilitation interventions remains a subject of debate within the medical community, accumulating evidence suggests that, for certain deficits, the initiation of rehabilitative protocols within the initial two weeks following a stroke event can yield discernible advantages. This reflects the existence of a temporally circumscribed phase characterized by augmented neuroplasticity in the early aftermath of a stroke, during which the cerebral response to injury is heightened, rendering rehabilitation interventions potentially more efficacious and impactful [22]. Potential benefits associated with early physical rehabilitation also include, improved muscle strength, physical function, quality of life, and reduced healthcare costs [23,24]. By shortening the onset to the initiation of rehabilitation, outpatient rehabilitation can bring real benefits to patients and thus succeed in providing treatment during the optimal window of opportunity following the stroke or acute hospitalization.

To conclude this chapter, the findings indicate that earlier initiation of rehabilitation is associated with reduced lengths of stay in inpatient facilities. This connection underscores the importance of timely rehabilitation, as it facilitates faster patient recovery and alleviates the burden on inpatient care by freeing up essential resources for other patients.

### 3.4. Intensity and Optimal Timing of the Outpatient Rehabilitation Program

A less intensive form of outpatient services is typically delivered following inpatient or home-based subacute rehabilitation programs. It is characterized by the involvement of fewer disciplines and is mostly self-managed by patients or their families rather than a rehabilitation specialist. In contrast to early primary programs, the efficacy of such services seems less projected and less documented. A systematic review that examined such rehabilitation services one year after a stroke could not indicate their effect on patients or families [25]. A systematic review of such outpatient services for chronic spinal cord injury patients conducted in 2005 in the Netherlands stated that it was impossible to conclude the positive effects of outpatient services [26]. A more recent systematic review from 2013 compared the benefits of different outpatient programs and found no significant difference between them [27].

The initial investigation for this project identified papers that compared various outpatient programs [28,29,30]. However, most papers compare these programs based on their settings rather than individual characteristics. Similar to various facets of the rehabilitation process, the key elements contributing to the success or failure of a rehabilitation program have been amalgamated within the broader “rehabilitation package”. This amalgamation complicates the task for decision-makers in discerning “what works?”, “for whom?”, and “when does it work?”

### 3.5. The Types of Outpatient Rehabilitation Settings

In 2019, the European Society of Physical and Rehabilitation Medicine (ESPRM) and the European Union of Medical Specialists—Physical and Rehabilitation Medicine Section (UEMS-PRM) convened a large team to develop clinical assessment schedules within the European framework of rehabilitation service types [12]. The professional medical rehabilitation community categorized outpatient rehabilitation services into several types: General and Specialized Outpatient Rehabilitation, General and Specialized Day Rehabilitation, Rehabilitation in Health Resorts, Rehabilitation in Primary Care, Home Rehabilitation Services, Rehabilitation for Specific Disabled Populations, and Community-Based Rehabilitation.

Each category was given a brief description. For instance, General Outpatient Rehabilitation involves patients living in the community who have various health conditions and are referred to an outpatient facility either after an acute or post-acute situation or by a community physician. Similarly, General Day Rehabilitation caters to patients requiring intensive treatment in a facility that accommodates them during the day and patients following an acute or post-acute situation or a referral from a community physician. Rehabilitation Services at Home assist patients who cannot access services elsewhere due to significant functional impairments, such as those with mobility issues or lack of transportation [15].

Day rehabilitation is often regarded as the most professional outpatient setting due to its high intensity and multidisciplinary approach, closely resembling inpatient programs. However, the research literature does not provide evidence supporting its superiority. A systematic review conducted in 2010 found that rehabilitation outcomes for acquired brain injury survivors in day hospitals were comparable to those from other in-clinic outpatient services [31]. However, they emphasized that most studies have compared novel interventions provided in community settings to standard care typically received. This means that the focus was not on the settings but on the differences in therapists, content of care, duration, and intensity of the programs. In essence, they were comparing fundamentally different approaches.

Home-based rehabilitation programs generally feature lower treatment intensity due to organizational constraints, making them suitable for patients with mild injuries or those who have difficulty leaving home. Despite these limitations, they can still be effective. A systematic review and meta-analysis published in 2022 from Hong Kong assessed the effectiveness of various outpatient service variables on the upper limb function of stroke survivors. The review found that home-based services achieved better outcomes compared to other community settings, particularly when supplemented with in-house assistive technology [31].

Monotherapy rehabilitation clinics, led by a rehabilitation doctor or one of the team professionals, can also provide an appropriate solution for some types of patients. An observational study that examined pulmonary outpatient rehabilitation in a supervised clinical setting vs. unsupervised home exercises found both beneficial but trended in favor of the supervised in-clinic form of rehabilitation [32].

### 3.6. The Value of Multidiscipline Care and Intensity of Therapy

In medical science, it has long been recognized that achieving favorable outcomes in rehabilitation requires integrating diverse elements. A crucial aspect involves orchestrating a multidisciplinary team of healthcare professionals possessing a broad range of clinical skills and specialized knowledge. The conceptualization and implementation of effective team configurations and collaborative efforts stem from the foundational belief that the interplay and synergistic interactions among team members can generate outcomes surpassing the mere additive effects of individual contributions [33].

Among physicians specializing in PRM, a prevailing consensus asserts that the favored paradigm for collaborative teamwork resides in the adoption of an interdisciplinary approach [34].

In the majority of healthcare environments, an interdisciplinary framework emerges as the most efficacious strategy, primarily due to its capacity to engender a cooperative, comprehensive, and patient-centric approach to the rehabilitation process [35]. The embracement of flexible, slightly interwoven delineations among professional roles expedites the flow of information, thereby enabling swifter interventions, and has been empirically demonstrated to hasten the discharge process [36].

Enhanced functional outcomes and, in some cases, even heightened survival rates can be realized through the employment of interdisciplinary collaboration across a spectrum of medical conditions. Particularly robust substantiation of these advantages can be found in the domain of stroke management, as documented in a Cochrane review [37]. Interdisciplinary collaboration is now essential to quality stroke services, as seen in inpatient rehabilitation.

These strategies were implemented in non-inpatients as well. The involvement of a multidisciplinary team and intense care seem to benefit neurologically affected patients mostly in terms of quality of life rather than in improving impairments and function. A Cochrane systematic review published in 2007 [38] found that a focused multidisciplinary intensive intervention has contributed to the quality of life and participation of patients with multiple sclerosis both in the short and long term. The measurements were compared to the regular outpatient services described in the papers that were included. As for stroke survivors, although a systematic review from 2013 [39] did not compare this intervention to any other, it nevertheless found the quality of life benefits in this population.

A systematic review of chronic back pain outpatient rehabilitation services was performed in 2002 in Toronto, Canada. It included 10 RCTs and found that intensive, multidisciplinary outpatient rehabilitation with a biopsychosocial approach was more effective regarding functional improvement and pain reduction than other forms of outpatient rehabilitation with fewer disciplines [40]. A year before, another systematic review examined how the same services affected neck and shoulder pain. The reviewers only identified two studies that have shown moderate evidence for the superiority of a multidisciplinary approach [41].

The advantage of engaging in a multidisciplinary program compared to a more targeted therapy program in outpatient rehabilitation outcomes appears to diminish in cases involving post-orthopedic operations, such as knee and hip arthroplasties [42,43]. A multidisciplinary approach becomes especially crucial when, in addition to the primary diagnosis, other meaningful contributing factors may significantly impact the rehabilitation outcome [32].

### 3.7. The Importance of a Professional Program Coordinator and PRM Physician Involvement

PRM represents a specialized medical discipline dedicated to advancing physical and cognitive aptitude, engaging in activities (inclusive of behavioral aspects), active participation (encompassing quality of life), and manipulating personal and environmental variables. Consequently, PRM assumes a pivotal role in the prevention, diagnosis, therapeutic intervention, and comprehensive rehabilitative oversight of individuals afflicted by debilitating medical conditions and coexisting comorbidities, spanning across the entire age spectrum [34].

According to a review by Singh et al. [44], in the realm of specialized rehabilitation, the leadership of a dedicated interdisciplinary team is ideally entrusted to a specialist in PRM. Traditionally, physicians have primarily led healthcare systems, with PRM training providing physicians with a comprehensive set of rehabilitative and medical skills. This training enables them to understand the full range of a patient’s impairments and functional limitations. The broad knowledge acquired by PMR physicians to effectively utilize the collective skills of the entire team, exert influence, and offer strategic guidance in patient care and progression. Crucially, within most nations’ professional and legal norms, the ultimate responsibility for a patient’s well-being rests squarely with the physician. This care model emphasizes that the physician should retain the overarching responsibility for decision-making within the team, as they must be prepared to justify and defend each decision. However, it is essential to note that in certain countries or settings, particularly in community-based contexts, the presence of a physician within the rehabilitation team may be lacking. In such instances, the ultimate responsibility for team decisions would typically fall on the team’s most senior member.

Effective leadership is a crucial prerequisite for success within interdisciplinary healthcare teams. A proficient leader must showcase various skills, including attentive listening, adept problem-solving capabilities, a proclivity for proactive managerial approaches, and a disposition amenable to conciliation. Additionally, recognizing, appreciating, and fostering individual disparities should be considered pivotal components of effective leadership. By employing collaborative methodologies, achieving consensus among team members should be an attainable and overarching objective [45].

We identified no studies attempting to quantify the value of an outpatient program coordinator, whether they were a PMR physician or another professional, in comparison. Nevertheless, the prevailing obstacle to the success of such programs appears to be the lack of communication and coordination among different disciplines, emerging as a significant theme in implementing these initiatives.

A systematic review published in 2014 [46] covered the determinants of the successful implementation of home-based rehabilitation. Of seven studies that met inclusion criteria, the author identified inter-professional collaboration and coordination of services as important determinants among a few others. Findings linked the coordination of services to a successful transition from hospital to home settings and continuity of care. Both require a case manager and a multidisciplinary team.

The need for a ‘case manager’ to coordinate the activities of the different disciplines in home-based rehabilitation services was strongly expressed by healthcare professionals who participated in a focus group in 2019. Examples of such statements were *“everybody holds a piece of the elephant, but nobody sees the whole picture”* (neuropsychologist, outpatient rehabilitation), and *“within primary care professionals simply do not run into each other. We need to organize inter-professional contact to be able to collaborate”* (manager in allied healthcare, primary care) [47].

Another qualitative study that focused on the challenges of outpatient cardiac rehabilitation [48] found poor coordination as a main problem. The interviewed program coordinators indicated that resource limitation prevented them from investing enough time in communication, is problematic, and may cause mistakes in patient management, which in its turn may increase the risk for patients’ health-related issues.

### 3.8. Clinical Communication in Outpatient Rehabilitation

In rehabilitation medicine, the efficacy of clinical communication stands as a linchpin for the success of patient treatment and overall outcomes. It serves as the bedrock for establishing trust, nurturing collaboration, and empowering patients throughout their journey to recovery [49].

Communication commences with the initial assessment, where clinicians prioritize active listening to grasp the patient’s concerns, aspirations, and expectations. As the rehabilitation journey unfolds, clear and concise communication becomes paramount, facilitating the conveyance of treatment plans, elucidation of exercises, and education on the patient’s condition and progress. Today, communication is acknowledged as a fundamental component of clinical interventions, shaping the development of a therapeutic patient-health professional relationship [50]. Over the years, research has consistently demonstrated that effective communication positively affects patient adherence to treatment, well-being, clinical biomarkers, and healthcare system costs [49,51].

Clinicians must tailor their communication styles to cater to each patient’s unique needs and preferences through verbal elucidation, visual aids, or written directives [52]. Although less frequent, outpatient communication remains pivotal for care, coordination, and progress monitoring. Clinicians must adeptly communicate treatment plans, exercise regimens, and self-management strategies during scheduled appointments. Patient education assumes a central role, as individuals bear responsibility for implementing treatment directives beyond clinic hours.

Moreover, effective communication in rehabilitation practice goes beyond patient-clinician interaction and encompasses interprofessional communication (IPC) [53]. IPC is essential for delivering cohesive patient care in rehabilitation settings, but it can be time-consuming, effortful, and occasionally frustrating, which may result in errors. Healthcare professionals should recognize that time dedicated to team communication is vital to patient care. It underscores the importance of effective communication strategies in rehabilitation settings. Rehabilitation teams are encouraged to reevaluate resource allocation for communication tasks and explore methods to improve communication efficiency, thereby saving time, reducing errors, and minimizing unnecessary repetition [54].

While essential in all rehabilitation settings, outpatient treatment presents unique challenges. Unlike inpatient care, which mainly serves patients with prolonged recovery periods and greater levels of impairment, outpatient IPC operates in a different context. In the inpatient setting, IPC tends to be more frequent and comprehensive, owing to the proximity between patients and healthcare providers. IPC rounds and team meetings foster real-time discussions regarding patient progress, objectives, and care strategies. Clinicians benefit from ample opportunities for direct observation and hands-on patient engagement, facilitating prompt feedback and adjustments to treatment protocols.

One of the distinct cases of IPC in outpatient rehabilitation is the communication between the rehabilitation team, particularly the PMR specialists, and primary care physicians. Clear communication pathways between them are indispensable for streamlining referrals, exchanging vital information, and maintaining continuity of care across diverse settings.

Families and other caregivers play an essential role in rehabilitation success. The caregiver might be beneficial or harmful to the patient’s functional progress, depressive symptoms, and marital relationship, depending on the rehabilitation phase [54]. Therefore, the involvement of families looms larger in inpatient rehabilitation, necessitating effective communication with caregivers. Studies emphasize the importance of meeting caregivers’ information needs, providing detailed and empathetic communication, and involving them in rehabilitation [55]. Communication with caregivers is crucial for ensuring seamless continuity of care and effective discharge planning.

Lastly, technology is increasingly pivotal in augmenting clinical communication within outpatient rehabilitation. In a recent publication, Goldman et al. [56] found that patient care delivered by multidisciplinary care teams through telehealth was effective, as patients appreciated the integrated approach, multiple perspectives, and effective communication within the team. Telehealth platforms serve as conduits for remote consultations and follow-ups, affording patients convenient access to care while upholding effective communication between appointments. Adopting telehealth could help reduce disparities between patients from different socioeconomic backgrounds, potentially improving healthcare access and outcomes for underserved populations [57].

In summary, the efficacy of clinical communication in outpatient rehabilitation is indispensable, influencing every aspect of patient care and interprofessional collaboration, and is continually evolving with technological advancements to better meet the needs of diverse patient populations.

## 4. Discussion

### 4.1. The Types of Outpatient Rehabilitation Settings

Our review yielded several significant findings. Various outpatient rehabilitation settings are available, encompassing general and specialized outpatient rehabilitation, day rehabilitation, community-based rehabilitation, and home-based rehabilitation programs [12]. Day rehabilitation is considered the most professional type of outpatient setting, similar to inpatient programs in terms of high-intensity and multi-professional treatment. However, evidence supporting the superiority of day hospitals over other outpatient services is lacking.

Outpatient rehabilitation programs are crucial in alleviating the strain on inpatient wards. In facilities with high demand, patients often face prolonged waits for hospital beds. Numerous reviews have highlighted the negative consequences of delays in the rehabilitation process, underscoring the importance of initiating rehabilitation at an earlier stage rather than later [31,40,41].

Starting rehabilitation earlier is crucial as it can significantly alleviate the strain on inpatient wards by promoting quicker recovery times. By initiating rehabilitation promptly, patients may experience improved outcomes that contribute to shorter lengths of stay. This frees up beds for new patients and enables healthcare providers to allocate resources more effectively, ultimately enhancing the quality of care across the system.

### 4.2. Outpatient Rehabilitation Cost-Effectiveness and Patient’s Satisfaction

Despite their lower treatment intensity, home-based rehabilitation programs can be effective, especially for patients with mild injuries or difficulties leaving their homes. Monotherapy rehabilitation clinics, led by PMR physicians or a team professional, can also provide appropriate solutions for specific patient types.

As demonstrated in systematic reviews, outpatient rehabilitation programs are generally more cost-effective than inpatient programs, leading to cost savings in health and social services [17].

Furthermore, patient satisfaction with outpatient services as a substitute for inpatient hospitalization is generally high. Research has shown that over 70% of patients express satisfaction with such services [18]. Patients appreciate the opportunity to receive multidisciplinary care in their homes, creating a more comfortable and familiar environment. However, some criticism has been raised regarding the lack of interdisciplinary communication, which, according to numerous studies, is essential for successful home rehabilitation, particularly in improving patient quality of life. This was more evident in patients with multiple sclerosis and stroke survivors but less so in post-orthopedic operations such as knee and hip arthroplasties.

The intensity and timing of outpatient rehabilitation programs play a crucial role in their effectiveness. Less intensive outpatient services, typically delivered after inpatient or home-based subacute rehabilitation programs, have shown less projected and documented efficacy. Studies have been inconclusive regarding the effects of these outpatient services, particularly for stroke survivors and spinal cord injuries. Further research is needed to determine the optimal intensity and timing of outpatient rehabilitation programs.

### 4.3. The Value of Multidiscipline Care and Intensity of Therapy

As previously noted, rehabilitation is inherently multifaceted and multifactorial [33,35,38,40,41,42,43,44]. Given its complexity, it necessitates comprehensive assessment, evaluation, and research as a multifaceted intervention [58]. Based on the results of the studies above, an iterative data aggregation and application process is essential to refine and enhance outpatient rehabilitation. In order to allow its adaptation and optimization, rigorous research should be conducted, and the results should be rapidly implemented in practice. For example, the insights garnered from patients and healthcare professionals regarding the lack of service coordination should be actively communicated [33,40]. It is recommended that a PMR physician be appointed to oversee these programs and reassess their effectiveness post-implementation. A thorough comparison of the different services should be conducted after each has been optimized to its full potential. Ultimately, rather than generally prioritizing one service over another, it is imperative to identify and consider factors that enable the customization and individualization of the appropriate program for each patient [59]. This information is crucial and can be a powerful tool for the patient’s case manager.

### 4.4. Clinical Communication in Outpatient Rehabilitation

Based on the research findings [49,50,51,52,53,54,55,56,57], effective clinical communication in outpatient rehabilitation is crucial for successful patient treatment and outcomes. It forms the foundation for building trust, fostering collaboration, and empowering patients throughout their recovery journey. From the initial assessment to the implementation of treatment plans, clear and concise communication ensures that patients understand their condition, exercises, and progress. The research underscores that effective communication positively impacts patient adherence, well-being, clinical biomarkers, and healthcare costs [49,51,52].

IPC is also vital in rehabilitation settings, especially in outpatient care, where the coordination between rehabilitation teams and primary care physicians is essential for seamless referrals and continuity of care. Although research indicates that IPC can be challenging and time-consuming, it is integral to cohesive patient care, necessitating efficient communication strategies to minimize errors and redundancy [53].

Furthermore, engaging families and caregivers through effective communication enhances rehabilitation outcomes, particularly in inpatient settings [55]. Caregivers’ involvement provides emotional support and facilitates adherence to rehabilitation protocols, which is essential for optimizing patient recovery [54]. Patients who receive active support from their families demonstrate improved motivation and engagement in their rehabilitation programs. Additionally, incorporating caregivers into the rehabilitation process can lead to more tailored and individualized care, as they can provide valuable insights into the patient’s home environment and daily challenges [36]. This collaborative approach empowers caregivers and ensures continuity of care beyond the inpatient setting, ultimately contributing to better long-term outcomes. Therefore, fostering strong communication channels with caregivers is essential for maximizing the effectiveness of rehabilitation interventions and enhancing overall patient well-being”.

Technology, such as telehealth, is increasingly important in supporting clinical communication, offering remote consultations and follow-ups that improve access to care and support interdisciplinary collaboration [56,57].

### 4.5. Limitations

Our review is subject to several limitations. First, the narrative review methodology itself has inherent limitations. The subjective nature of selecting and interpreting studies may affect the comprehensiveness and objectivity of the review, and only studies available in English were included. Additionally, the lack of a formal quality assessment for the included studies may impact the overall validity of the conclusions drawn.

Despite diligent efforts to locate pertinent studies on the given topic, only a limited number were identified, with an even smaller subset being RCTs. This scarcity suggests that the subject has not been extensively investigated, emphasizing the need for additional research to achieve a more definitive conclusion. For example, the role of a professional program coordinator in outpatient rehabilitation has not been extensively studied. However, the presence of a case manager or program coordinator is crucial for effective coordination and continuity of care.

### 4.6. Recommendations for Further Research

Further research should concentrate on the following aspects: More studies are needed to investigate the role of a professional program coordinator in outpatient rehabilitation to understand the impact on effectiveness and continuity of care. Identifying factors that can tailor and individualize the right program for each patient is crucial to maximizing each service’s potential. Additionally, research should explore the role of effective clinical communication in outpatient rehabilitation, understanding how clear and concise communication impacts patient adherence, well-being, clinical biomarkers, and healthcare costs. Investigating the role of technology, such as telehealth, in supporting clinical communication and interdisciplinary collaboration is also essential. Understanding how technological advancements can reduce disparities and improve healthcare access and outcomes for underserved populations is critical.

## 5. Conclusions

Non-inpatient rehabilitation programs have shown cost-effectiveness and high patient satisfaction compared to inpatient programs. However, the optimal intensity and timing of these outpatient services require further investigation. Various outpatient rehabilitation settings exist, each with unique advantages and considerations. Multidisciplinary and intensive rehabilitation can significantly improve patients’ quality of life, though their effectiveness may vary by condition.

The success of outpatient rehabilitation programs heavily depends on the intensity and timing of interventions. High-intensity, multidisciplinary programs, especially when delivered promptly after inpatient or home-based rehabilitation, are more likely to enhance patient outcomes.

One critical aspect that emerged from this review is the importance of having a dedicated program coordinator, such as a PRM physician. This role is crucial for ensuring continuity of care and effective communication among the healthcare team. Although the impact of this position has not been extensively studied, its presence is essential for the success of rehabilitation programs. Future studies should explore this role in depth to understand its full potential.

Effective clinical communication in outpatient rehabilitation is essential, impacting all facets of patient care and interprofessional collaboration. It continuously adapts and improves through technological advancements to serve diverse patient populations better.

In practical terms, healthcare administrators and policymakers should consider the abovementioned factors when developing and implementing outpatient rehabilitation programs. Emphasizing a multidisciplinary approach, ensuring the presence of a program coordinator, and fostering robust clinical communication can lead to more effective and sustainable rehabilitation outcomes. Furthermore, ongoing research is needed to refine these approaches and identify the most beneficial practices for specific patient groups.

By focusing on these key elements, outpatient rehabilitation programs can achieve greater efficacy and benefit patients recovering from various conditions, ultimately leading to improved health and quality of life.

## Figures and Tables

**Table 1:** Number of papers included in each chapter.

Chapter Number	Chapter Name	Number of Sources Used
3.2	Outpatient rehabilitation cost-effectiveness and patient satisfaction.	6
3.3	Reducing the load from the inpatient wards.	5
3.4	Intensity and optimal timing of the outpatient rehabilitation program.	6
3.5	The types of outpatient rehabilitation settings.	4
3.6	The value of multidiscipline care and intensity of therapy.	12
3.7	The importance of a professional program coordinator and PRM physician involvement.	6
3.8	Clinical communication in outpatient rehabilitation.	9
Total		48 *

* A total of 45 articles were used. Since 3 of them were repeated in different chapters, the total count came to 48.

## Data Availability

The data supporting this study’s findings is available from the corresponding author upon reasonable request.
